# Extermination of the Cattle-tick1Read before the Virginia State Veterinary Medical Association at Norfolk, June 24, 1896.

**Published:** 1896-09

**Authors:** Cooper Curtice

**Affiliations:** Moravia, New York


### THE JOURNAL OF COMPARATIVE MEDICINE AND VETERINARY ARCHIVES.

VOL XVII. *SEPTEMBER*, 1896. No. 9.

### ON THE EXTERMINATION OF THE CATTLE-TICK AND THE DISEASE SPREAD BY IT.[1]

BY COOPER CURTICE, D.V.M.,

MORAVIA, NEW YORK.

I AM honored by your President in being invited to address you upon one of the two most important epizoötic diseases now to be found among cattle in the United States. I prefer to present it to you under the name "tick fever," as the tick, *Boophilus bovis*, is as far as is yet known the most important, if not the only, factor in its transmission. You will recognize by this name that disease to which more terms have been applied than to any other—a fact which indicates the lack of precise knowledge concerning it held either by the laity or profession, viz.: Southern cattle fever, splenetic fever, Spanish cattle fever, Texas cattle fever, Carolina cattle distemper, Mexican or Indian cattle disease, distemper, bloody murrain, red-water, hæmaturia, splenic fever, hæmoglobinuria, tick fever, acclimatization fever, etc.

It is the relationship between the tick and the disease, and the means of eradicating them from Virginia and the United States, to which I invite your attention. Repeated experiments by the U. S. Department of Agriculture, by your colleagues Harbaugh and Niles, of this State, by Dinwiddie, of Arkansas, and on a larger scale by the stockmen of the country, have shown conclusively that the tick does give the disease to susceptible cattle. Among all other experiments made to demon-

[1] Read before the Virginia State Veterinary Medical Association at Norfolk, June 24, 1896.

strate another mode of infection, not one has succeeded, excepting the direct transmission of blood from previously infected but healthy cattle to susceptible cattle. Since this mode of infection is necessarily rare and not possible except by human agencies, it may be passed by as of secondary importance in the transmission of the disease.

Permit me to repeat, ticks are the only known means of spreading the disease. If so, is it not self-evident that to prevent the disease spreading the distribution of the tick must be further restricted and finally the species exterminated?

In the report of the U. S. Department of Agriculture on Texas fever, 1893, the question as to there being another mode of infection in those States where the disease is more or less permanently located is left an open one. Nor can it be settled until some territory is found in which the cattle are free from ticks, and in which cattle brought there die of the disease without having been infected by ticks. When such a place is found the study of the disease may be taken up just where it has stopped, and new causes or methods of transmission found. This leads us to the question of climatic, telluric, or other conditions which may be supposed to foster and spread the disease aside from the parasite, which we know distributes it far and wide.

The prevalence of the disease in the Southern States in spring, summer, and autumn, among cattle which are merely moved from farm to farm, or, at the most, not more than a few miles, is a strong argument that neither climate nor soil has aught to do with the spread of the disease, for they are the same. Mr. A. W. Bitting, in Bulletin No. 28 of the Florida Agricultural Station, 1894, describes an outbreak in a herd of cows, bred by the owner near Tallahassee, and kept sound until moved to a tick-infested farm near-by, where the "land and water were about the same," and all became affected.

In Georgia there are enormous annual losses from red-water among cattle which are merely moved about in neighborhoods, and farms are classed as infected although their soil and water privilege are identical with neighboring places where no native cattle are lost but where cattle die when moved.

In your own State there are adjoining counties in which murrain is known or not known; farms which have never lost cattle, while other cattle brought to them or taken from them frequently die. Nor is it necessary to move them from a highland to lowland or from the north to the south. The movements may take place in any direction, but the results will be the same.

In this disease two classes of cattle must be constantly borne in mind—those that have become unsusceptible and those that are susceptible. The death-record in infected localities indicates the presence of infection and of susceptible cattle, and when they are raised near-by that the soil and climate, which is the same for all, have no more immediate relation to the disease in Florida than it would have on neighboring farms in New York. When spread the disease will break out in either; when confined there will be no outbreak, although the means of spreading be close at hand in both.

Counties and farms in this and other States have been infected and reinfected, and are to-day as free from disease as any in Maine, though south of or north of or east of or west of others, or higher in altitude than others, or lower than others. and containing more or less bottom-lands than others, and growing more or less pine or oaks than others, and watered by standing water, running water, etc.

Further, when outbreaks are closely scrutinized the pestiferous tick is always found. In the murrain counties, Buckingham, Patrick, Pittsylvania, Gloucester, Lunenburg, and others, cattle-ticks abound. When Prince Edward County before the war lost her cattle by murrain the ticks, I am informed, were there. Since then they have disappeared, and cattle are no longer killed by them, excepting as farms are reinfected, as may occur anywhere.

If you will ride with me east, west, north, or south through this State and inspect farm after farm and make inquiries, you can come to no other conclusion than that this disease of many aliases is coincident in distribution with the tick, irrespective of altitude, topography, drainage, soil, food, verdure, or climate, Some or all of these factors may influence the spread of the tick, but not the coincidence of the tick and the disease.

Let me explain what I mean by disease in this instance. I include not only the paroxysm of fever that an animal may have, but the changed condition of its system which permits it to raise the ticks with impunity. In this sense a cow perfectly healthy for all other purposes might contain blood which at any time would disease others under favorable conditions. All blood for this purpose must be considered in a pathologic condition; it is altered from the normal. There is then, so far as is yet known, nothing in the climate or soil of the so-called permanently infected country which we may consider causative of the disease.

Both history and experiment indicate the contrary. In the sixteenth, seventeenth, and eighteenth centuries this country was colonized by two classes of people, who brought their cattle with them. You well know that the Spanish colonized the West Indies, Florida, Mexico, Texas, New Mexico, and California, and the northern European nations the thirteen colonies. The Spanish endeavored to push northward along the Atlantic coast, or at least to hold the southward advancing pioneers in check. Until the cattle of the Spanish began to mingle with those of the English to the north, those of the latter prospered and multiplied. After that cattle died from tick-fever, and have continued to die in numbers proportional to the movement and number of cattle involved. This occurred as early as, if not earlier, than the eighteenth century. I believe that Carolina cattle were infected from Florida or Cuban cattle. Virginia cattle were certainly infected by Carolina cattle, and also, I believe, by cattle introduced by coastwise traders, who traded in barter between Cuba and other West Indian Islands and Maine. The disease had become so prevalent in your State in 1750 that special legislation directed against it was necessary. This legislation has been amended from time to time, an amendment being adopted at the last session of your Legislature.

As the Spanish cattle spread over Texas, and the great markets of the North, about 1840 or even before, began to require beef, the Texans were driven over trails northward, spreading disease along their route. The disease from these centres slowly spread unhindered by law or man until it was checked by the laws of climate, which restricted the spread of the disease. The Spanish invader of this continent was responsible for many things. Could he have planned as diligently as he did inquisition methods to have left behind him a cattle-plague which would harass his foeman for ages, he could scarcely have found a better means than the tick.

The experiments of the Bureau of Animal Industry show as conclusively as may be that no disease-giving property is communicated by cattle to the ground. The matter up to now then lies this way. This cattle-plague is nothing more or less than the result of an invasion of this continent by the parasitic tick which cattle carry with them.

Is this invasion permanent or temporary? Mr. H. J. Wing, of the Experimental Station in Georgia, picked and killed every tick on his cows in 1892. Since then he has had none. Although he bought ticky cows, he confined them in the stable until he had cleaned them. On that farm cattle from all parts are mingled and none die. Last year he cleaned an adjoining farm of ticks by regular hand-picking, permitting none to escape. Although the cattle went to pasture daily, the process occupied but little time, three times a week for a couple of months, although the observation was continued longer.

The invasion of these farms was, therefore, but transient. The invasion of many counties and farms in Virginia, although well within the permanent area, is well-known to have been transient. There is no farm in this State or any other State but that is transiently infected by ticks. The word permanent as it relates to ticks has no place in the American vocabulary. All ordinary herds may be freed with comparatively little trouble by confining animals and hand-picking. If they are kept on an enclosed farm, regular, persistent hand-picking will free them. And the labor is not so great as it seems. If cattle roam upon commons, then all owners must keep their own cattle free. This will be found to be the most effective means for freeing all cattle in Virginia. If, however, enclosed land is abundant, the ticks may be kept from cattle by picking off those on them at the time and then turning the latter on to uninfected pastures hitherto unused by cattle. Thorough greasing of the hairs by cotton-seed oil, lard, or kerosene will prevent the little ticks arriving at the skin. Destruction of the ticks by burning the land in spring or by plowing will also assist the hand-picking. In Virginia and most other States I believe that there is little economy in dipping cattle. Nearly all herds are too small to permit the outlay. Where it must be resorted to those practising it must know that the operation must be carried on regularly, as often as need be, until it is quite sure that the cattle pick up no more on the pastures.

To restrict the advance of the disease northward in summer the United States annually quarantines the cattle from certain Southern States or portions of them, not permitting their transportation northward, excepting under regulation which keeps them from other cattle. This quarantine, if as effectually carried out in other States as it is now in Virginia, is very efficacious. But the United States should not seek to perpetually quarantine, but to contract the quarantined, and thus lessen the menace at every point. It has shown that the means of transmission is a remediable one. Let it do its part in applying the remedy. It is clearly within its province to continually investigate the border-line between the quarantined and free territories, and proceed to learn the exact conditions of infection, to study the effect of local laws and customs upon its spread, to instruct the cattlemen at certain points as regards the suppression of the ticks, and to change the line in accordance with the facts and needs arising.

Whatever the future may show as to there being another means of producing the disease in the South, the Bureau of Animal Industry has shown that as soon as the ticks are removed from sections of country the cattle from these places are no menace to others, and, therefore, that the quarantine-line must be, in so far as it is practical to conform to political boundaries and natural barriers, coincident with the edge of the region infected by these pests. If for no other reason than that of uninterrupted traffic, the cattlemen of the infested region should make haste to exterminate the tick.

The Legislature of this State at its last session passed a Contagious Disease Act, which will prove very efficacious in preventing the spread of the disease in the free territory. It may apply to the uninfected territories within the quarantined region, but the fines are so heavy that complaints will scarcely be made. For instance, in those localities cattle with ticks are virtually quarantined upon their owners' farms, for there is a fine of from one hundred to five hundred dollars for permitting them upon the highway or commons.

Could the quarantine of ticky cattle upon the infected farms be rendered effective, the great question for Virginia would be more than half settled, for no owner would permit his cattle on the highway before they were cleaned of ticks, and, being in constant danger from the ticks spreading from his farm, he would be induced to remove them. When the State is ready to remove the ticks farm quarantine must be resorted to. I believe that at present a light fine of from two to five dollars per head for cattle with ticks found on the highway, unenclosed lands, or commons, and impounding them until the cattle are cleansed and the fine paid, would be far more efficacious than the high fine imposed by the present law and which is necessary to prevent violations of the State quarantine-line.

Your duties in this instance are to do all in your power to keep up a perfect quarantine between the territory partially infected and the free district. Those of you who live within the infected district owe it to your patrons to instruct them as to how the disease is spread, to show them, even if it is necessary to take some farm as an experiment, how to rid that farm of its ticks, and, more than all, to keep the public market-places and cattle ranging at large free from ticks. I have found that cattle-owners are only too willing to fall in line when once they are convinced that it is of use. You should use every means at your command to show its usefulness.

By means of the wide-awake Virginia veterinarians I believe that in less than five years tick-disease or murrain in this State will be an unknown quantity. You owe it to yourselves and to your fellows in other States to show what may be done by a few willing, courageous men. The task is a huge one, but if you do what you find to do, it will be accomplished.

I look most eagerly for the cleansing of even a certain portion of the infected territory under the direct intention of man, for it opens the way to pushing the ticks back to the Spanish isles and Mexico, and liberating cattle from disease and pests and the farmer from untold money losses. Let your war-cry be, death to the tick.

